# Neuregulin‐1 is essential for nerve plexus formation during cardiac maturation

**DOI:** 10.1111/jcmm.13408

**Published:** 2017-12-19

**Authors:** Daniel Brown, Leigh Ann Samsa, Cade Ito, Hong Ma, Karla Batres, Rima Arnaout, Li Qian, Jiandong Liu

**Affiliations:** ^1^ McAllister Heart Institute University of North Carolina Chapel Hill NC USA; ^2^ Department of Pathology and Laboratory Medicine University of North Carolina Chapel Hill NC USA; ^3^ Department of Cell Biology and Physiology University of North Carolina Chapel Hill NC USA; ^4^ Department of Medicine Division of Cardiology Cardiovascular Research Institute University of California San Francisco CA USA

**Keywords:** neuregulin‐1, cardiac innervation, cardiac maturation, zebrafish

## Abstract

The Neuregulin‐1 (Nrg1)/ErbB pathway plays multiple, critical roles in early cardiac and nervous system development and has been implicated in both heart and nerve repair processes. However, the early embryonic lethality of mouse *Nrg1* mutants precludes an analysis of Nrg1's function in later cardiac development and homeostasis. In this study, we generated a novel *nrg1* null allele targeting all known isoforms of *nrg1* in zebrafish and examined cardiac structural and functional parameters throughout development. We found that zebrafish *nrg1* mutants instead survived until young adult stages when they exhibited reduced survivorship. This coincided with structural and functional defects in the developing juvenile and young adult hearts, as demonstrated by reduced intracardiac myocardial density, cardiomyocyte cell number, swimming performance and dysregulated heartbeat. Interestingly, *nrg1* mutant hearts were missing long axons on the ventricle surface by standard length (SL) 5 mm, which preceded juvenile and adult cardiac defects. Given that the autonomic nervous system normally exerts fine control of cardiac output through this nerve plexus, these data suggest that Nrg1 may play a critical role in establishing the cardiac nerve plexus such that inadequate innervation leads to deficits in cardiac maturation, function and survival.

## Introduction

While heart development is known to require coordination between multiple cell types 1, the complex genetic interactions governing coordination between the nervous system and the developing heart 2 are largely unknown and understudied. Obtaining a greater understanding of when and how Neuregulin‐1 (Nrg1)/ErbB signalling directs cardiac development and homeostasis will allow scientists and clinicians to harness this pathway for cardiovascular therapies. The Nrg1/ErbB2 signalling pathway has been strongly implicated in multiple aspects of development, homeostasis and disease in both cardiac and nervous systems 3, 4, 5, 6. Canonically, transmembrane pro‐Neuregulin‐1 (Nrg1) is cleaved to release Nrg1 ligands that interact with ErbB2/ErbB4 or ErbB2/ErbB3 receptor heterodimers, leading to tyrosine kinase phosphorylation events that promote downstream signalling with broad effects 4, 7. Several rodent studies have demonstrated that each component of the Nrg1/ErbB pathway is essential for cardiac chamber maturation during embryogenesis 3, 8, 9, 10, 11, and while embryos appear to die *in utero* from cardiovascular failure, these defects are coincident with defects in the developing nervous system 8, 10, 11. The zebrafish, *Danio rerio*, represents a premier model organism for studying the molecular and genetic regulation of the heart development, given their rapid external development, optical clarity and ease of genetic manipulation 12. Furthermore, although mammalian embryos rapidly deteriorate from cardiovascular defects, zebrafish are relatively hypoxia tolerant 13. Notably, heart development in zebrafish is not completed at embryonic or larval stages. For example, zebrafish hearts develop a critical outer layer of cortical myocardium about 6–8 weeks post‐fertilization, as young adults 14. Additionally, trabeculae continue developing through adulthood, and the adult myocardium is comprised primarily of an expanded and remodelled meshwork of trabeculae 15.

Nrg1 growth factors are known to be necessary for both central and peripheral nervous system development 16. With respect to cardiac innervation by the peripheral nervous system, axonal derived NRG1 signals *via* ErbB2 and regulates multiple aspects of Schwann cell migration and differentiation during development 17, 18, 19. Nrg1 has been implicated in axoglial signalling required for peripheral nerve development 16, 20, and there is strong evidence that Nrg1 is required for effective nerve regeneration and repair 21, 22. Furthermore, solubilized Neuregulin‐1 has demonstrated therapeutic effects on nerve regeneration and is currently under development as a therapy for heart failure 23, 24.

Our previous work and others have shown that ErbB2 and ErbB4 have conserved roles in formation of zebrafish cardiac trabeculae 25, 26. Nrg1 binds *via* its EGF domain to ErbB4 expressed on cardiomyocytes, promoting dimerization with the essential co‐receptor ErbB2. While ErbB4 binds the ligand, and has limited tyrosine kinase activity, ErbB2 has no ligand‐binding activity, but has kinase activity that is necessary to modulate cardiomyocyte gene expression 3, 4. Zebrafish *nrg 1* has three major isoforms produced by alternative splicing, *nrg1‐I*,* nrg1‐IIa‐c* and *nrg1‐III,* of which *nrg1‐I* is the primary isoform expressed in the developing heart 19, 27. Transmembrane pro‐Neuregulin‐1 (Nrg1) expressed on endocardial and/or microvascular endothelial cells is cleaved by proteases to release active Nrg1 3, 26. However, our previous study demonstrated that zebrafish deficient in *nrg1‐I* and *nrg1‐II* developed trabeculae in an ErbB2‐dependent manner and survived to reproductive adulthood with no overt cardiovascular defects 27. Despite the dispensability of *nrg1‐I* and *nrg1‐II* in trabecular development, Perlin *et al*. 19 demonstrated that loss of *nrg1‐III* in zebrafish caused defects in myelination due to impaired Schwann cell migration leading to supernumerary neuromasts in the developing larvae. While loss of *nrg1‐III* in zebrafish caused defects in myelination, there have been no reports examining cardiac consequences or innervation defects associated with total loss of Nrg1 function in zebrafish.

In this study, we use CRISPR/Cas9 targeted nuclease activity to target *nrg1* at an essential exon shared by all *nrg1* isoforms to make *nrg1* ^*−/−*^ zebrafish. While Nrg1 is dispensable for early cardiac development, our findings demonstrate an essential role for Nrg1 in contributing to proper cardiac nerve expansion and maturation at juvenile stages.

## Materials and methods

### Animal lines and care

All animals were raised and maintained at the aquaculture facility of the University of North Carolina at Chapel Hill in accordance with Institutional Animal Care and Use Committee approved protocols 28. The zebrafish lines used in this study are as follows: *nrg1* ^*nc26*^ *, nrg1* ^*nc27*^ *, nrg1* ^*z26*^ 19, *Tg(myl7:rasGFP)* ^*s883*^ 29 and *Tg(myl7:DsRed‐NLS)* ^*f2Tg*^ 30.

### CRISPR/Cas9 gene editing

*CRISPR/Cas9* targeting of *nrg1* at Exon 6 was performed essentially as previously described 27. Briefly, sgRNA target sites predicted to not bind off‐target sites in the exome were identified using ZiFit software and zebrafish genomic sequence build GRCz9. Cas9 mRNA and sgRNAs were *in vitro* transcribed then injected into WT (outbred TL strain) embryos at the one‐cell stage. Mutant alleles were confirmed by Sanger sequencing of PCR amplified genomic DNA spanning Exon 6 in F2 individuals.

### Neuromast development

Neuromast numeracy was assayed in live larvae, essentially as previously described 27, 31. Briefly, 4–5 days post‐fertilization (dpf) larvae from heterozygote interbreedings were anaesthetized and incubated in fish water containing Mitotracker Red (Thermo Fisher Scientific, Waltham, MA, USA). At least 16 individuals from *N* ≥ 3 clutches were imaged in the dorsal position using a Leica M205C fluorescence stereomicroscope. The number of MitroTracker Red stained neuromasts was manually counted from a one lateral line per individual.

### Genotyping

Genomic DNA from fin clips or embryos was used to genotype *nrg1* ^*nc26*^ and *nrg1* ^*nc27*^ fish by high‐resolution melt analysis (HRMA) as previously described 27. Primers used span the target site in Exon 6 (F – GTTGTGTTCCTCTGTGCAGC, R – TTCTCGCTCTCATTGCAGGG). For the survival curve (below), the genotype of mutant larvae 4–5 dpf was in inferred by neuromast supernumerary. These methods were verified both by digestion with BsaJI and Sanger sequencing.

### PCR and qRT‐PCR

PCRs were performed with genomic DNA or cDNA template as per manufacturer's instructions (GoTaq, Madison, WI, USA; SybrGreen, Thermo Fisher Scientific). RNA was isolated from adult hearts (Qiagen RNAeasy, Thermo Fisher Scientific) and up to 1 μg reverse transcribed into cDNA (Supermix III, Invitrogen) according to manufacturer's instructions. A ViiA7 qPCR machine was used for expression analysis where cycle threshold (CT) values were normalized to *ef1a* as a housekeeping gene, and relative expression was calculated comparing average change in CT in wild‐type and mutant samples by the 2^^(ΔΔCT)^ method (Livak and Schmittgen, 2001). Primers include Nrg1‐E6‐E10 F (CACTGCTGCTTTGTTGGACG), Nrg1‐E6‐E10 R (TGCTGTTCACTCAGTGGCAA), *ef1a* F (AGGACATCCGTCGTGGTAAT) and *ef1a* R (AGAGATCTGACCAGGGTGGTT).

### Larval trabeculation assay

Larvae were treated from 60 hpf with 3.5 μM of ErbB2 kinase inhibitor PD168393 or 1%DMSO vehicle alone. At 4 dpf, larvae were anaesthetized embedded and oriented in 1% low‐melt agarose and manually oriented. Z‐stacks of the middle of the ventricle were collected immediately post‐mortem (killed with Tricaine) using an Olympus Fluoview 1000MPE with 20× XLPlan water immersion objective (NA 1.0) and 2.5× optical zoom with at least 512 × 512 pixel resolution and 1–2 μm (20×) step sizes. Confocal data were collected for a minimum of three biological replicates for each condition. ImageJ 32 was used to select a single representative z‐plane for each animal.

For whole animal imaging, adult fish were anaesthetized and imaged in a minimal volume of water using an Android 13 MP camera. Brightness and contrast were adjusted, and images were scaled using ImageJ 32.

### Survival curve

Offspring from interbreeding *nrg1* ^*wt/nc26*^ fish were screened at 5 dpf for a supernumerary neuromast phenotype to separate *nrg1* ^*nc26/nc26*^ fish from *nrg1* ^*wt/wt*^ and *nrg1* ^*wt/nc26*^ clutch mates. Survival was recorded weekly from *N* = 7 tanks of 10 fish each over 12 weeks.

### Cardiac innervation immunostaining and quantification

Whole zebrafish hearts were dissected then stained with anti‐acetylated alpha tubulin (ACT) essentially as previously described 33. Juvenile hearts were too small to handle for histology‐hearts were partially dissected *in situ* for optical accessibility. Hearts from fish SL10‐SL20 were dissected and imaged directly. Due to atrial collapse post‐fixation, extent of surface innervation was quantified only on the ventricle. For juvenile and adult hearts, ventricles were oriented to the dorsal surface and imaged by confocal microscopy. Samples were embedded and oriented in 1% low‐melt agarose and manually oriented. Z‐stacks were collected using an Olympus Fluoview 1000MPE with 10× UMPLFN (NA 0.3) or 20× XLPlan water immersion objective (NA 1.0) and up to 2.5× optical zoom with at least 512 × 512 pixel resolution and 9 μm (10×) or 1–2 μm (20×) step sizes. Using ImageJ software (Schneider *et al*., 2012), ACT+ nerves were traced from the maximum project image and the extent of innervation reported as a ratio of the total length of axons relative to assayed surface area.

### Histology

Histology was performed essentially as previously described 27. Briefly, adult fish were fixed in 4% PFA then de‐calcified with 0.5 M EDTA for 3–7 days. Samples were either paraffin embedded and sectioned to 8 μm or frozen in OCT and cryosectioned to 10–14 μm. H&E‐stained tissues were imaged using a Leica DMIRB inverted microscope.

### Adult myocardial area, cellularity, and cell size measurements

To calculate the per cent area of the inside of the ventricle wall composed of cardiomyocytes and the number of nuclei within the area, fish expressing *Tg(myl7:rasGFP)* with or without *Tg(myl7:dsRed‐NLS)* transgenes were cryosectioned as described above. Confocal optical sections of the heart were obtained with a Z‐resolution of 9 μm using an Olympus Fluoview 1000MPE with 10× UMPLFN (NA 0.3) water immersion objective with at least 512 × 512 pixel resolution. Image J was used to measure myocyte area by setting threshold values to segregate myocardium pixels from background pixels in the outer wall of the ventricle. To count nuclei, a threshold value was established for the positive signal and the number of objects within the expected size range of a cross‐sectioned nucleus was counted. This methodology was validated in a subset of samples by manual measurements. To measure cell size, adult hearts from zebrafish SL15‐SL20 expressing *Tg(myl7:rasGFP)* were enzymatically dissociated into a single‐cell suspension essentially as previously described 34. Cells were centrifuged at 1200 g for 5 min. and imaged at 20× with conventional fluorescence microscopy (EVOS, Invitrogen). ImageJ software (Schneider *et al*., 2012) was used to measure the surface area in 200–250 mononuclear myocytes from at least *N* = 3 biological replicates for each genotype in three separate experiment.

### Electrocardiography

Electrocardiography was performed on *N* = 4 adult fish standard length (SL) 16–20 essentially as previously described 35. Heart rate (HR) and heart rate variability (HRV) were calculated using LabChartReader software (AD Instruments, Colorado Springs, CO, USA).

### Swim performance

Critical swimming velocity (UCrit) of adult *nrg1* ^*wt/wt*^ and *nrg1* ^*nc26/nc26*^ fish SL18 ± 1 was measured in a Loligo Systems (Viborg, Denmark) swim tunnel essentially as previously described 36.

## Results

### Zebrafish neuregulin‐1 and generation of nrg1 knockout

**Figure 1:**
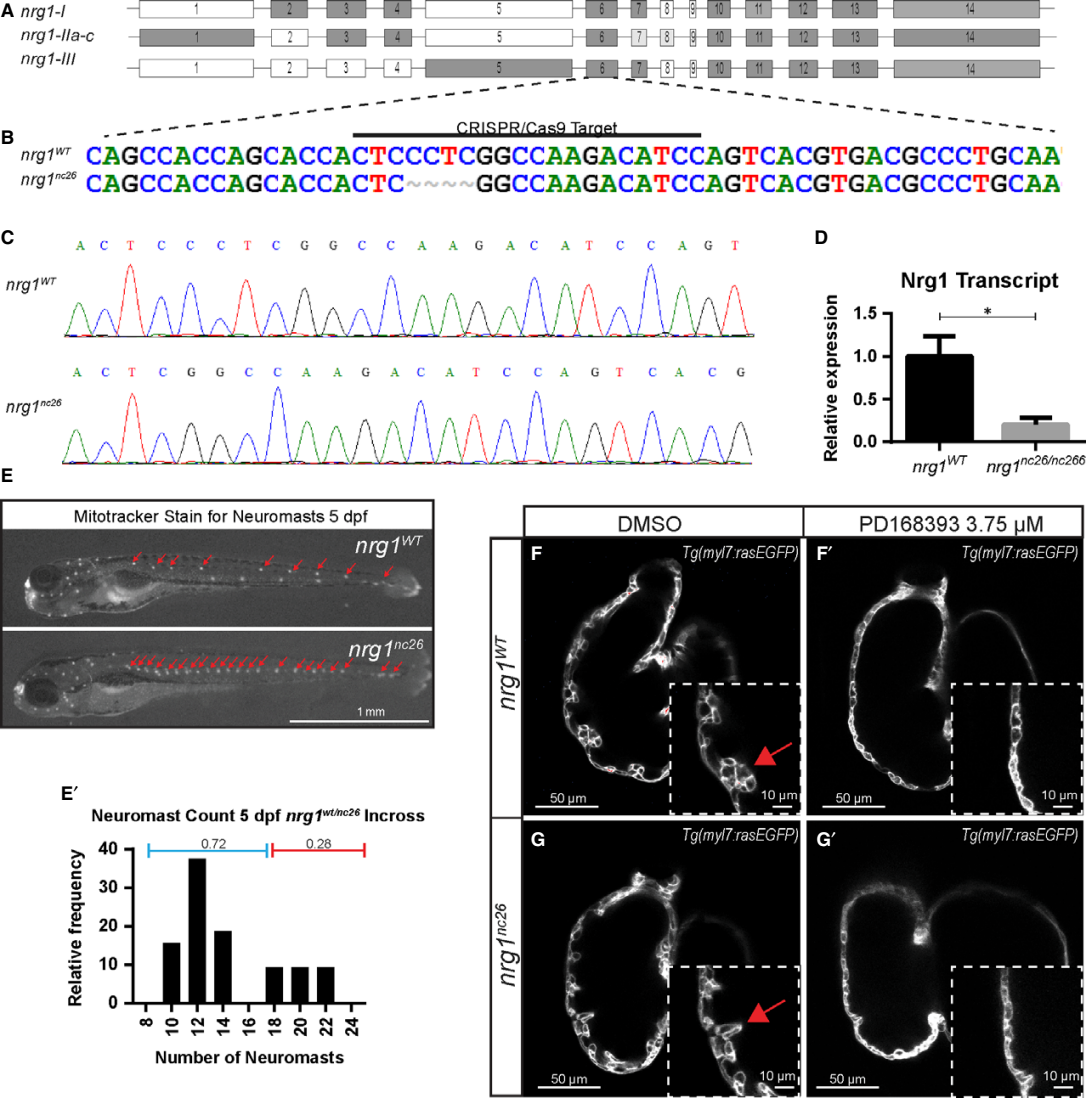
Generation and validation of *nrg1* ^*nc26*^ *allele*. (**A**) Gene structure of zebrafish *nrg1* (exons drawn to scale). Alternative splicing produces three primary isoforms, *nrg1‐I*,* nrg1‐IIa‐c* and *nrg1‐III*. (**B, C**) CRISPR/Cas9 targeting at Exon 6 produced the *nrg1* ^*nc26*^ allele with a four amino acid deletion. (**D**) Detection of *nrg1* mRNA transcripts spanning Exons 6–10 by qPCR; statistical significance determined by t‐test, *P < 0.05. (**E**) Representative *nrg1* ^*wt*^ and *nrg1* ^*nc26/nc26*^ clutchmates stained with Mitotracker Red to detect neuromasts in the developing lateral line at 4–5 dpf. Red arrows designate neuromasts. (**E’**) Relative frequency of the number of neuromasts counted in embryos from heterozygous incrosses of *nrg1* ^*wt/nc26*^ fish, *N* > 24 larvae. (**F‐G’**) Representative confocal optical mid‐chamber slice of the ventricle at 3–4 dpf in larvae carrying *Tg(myl7*:*rasGFP)* cardiomyocyte reporters and treated with DMSO or 3.75 μM ErbB2 inhibitor PD168393. Boxes include high‐resolution image of the outer curvature. Red arrows point to developing trabeculae.

The zebrafish genome encodes several members of the neuregulin family—*nrg1*,* nrg2a*,* nrg2b* and *nrg3*. Sequence analysis indicates zebrafish Nrg1 is the closest homolog to human NRG1 and mouse Nrg1 27. Alternative splicing of *nrg1* produces three main isoforms, *nrg1‐I*,* nrg1‐IIa‐c* and *nrg1‐III*, which differ primarily in their N‐terminal sequence and share EGF‐like, transmembrane and ‘neuregulin’ domains^7^, ^37^ (Fig. 1A). To investigate the requirements for any isoform of *nrg1* in heart development, we used CRISPR/Cas9 gene editing to target Exon 6 just upstream of the EGF‐like domain, shared by all three isoforms. We isolated two mutant alleles, *nrg1* ^*nc26*^ and *nrg1* ^*nc27*^ encoding four and eight base pair deletions, respectively (Fig. 1B and C, Fig. S1A and B). These mutations are predicted to encode frameshifts and early truncations within the EGF‐like domain (Fig. S2). In support of a true knockout, we observed a significant reduction in abundance of *nrg1* mRNA in *nrg1* ^*nc26*^ mutant hearts (Fig. 1D). To verify functional effects of the new mutations on *nrg1‐III*, we assessed whether *nrg1* ^*nc26*^ and *nrg1* ^*nc27*^ larvae share phenotypic abnormalities with *nrg1* ^*z26*^ homozygous mutants. In the *nrg1* ^*z26*^ allele, a single point mutation in exon 1 leads to loss of function of the CRD domain of Nrg1‐III. This is previously reported to impair Schwann cell migration, leading to supernumerary neuromasts in the developing lateral line 19. We used a voltage sensitive vital dye (Mitotracker) to label neuromasts in larvae produced from interbreeding heterozygous *nrg1* ^*wt/nc26*^ or *nrg1* ^*wt/nc27*^ fish. Supernumerary neuromasts were observed at Mendelian ratios (Fig. 1E; Fig. S1C). Sequencing of a subset of larvae that demonstrated greater than18 neuromasts verified these were homozygous for *nrg1* ^*nc26*^ or *nrg1* ^*nc27*^ alleles (data not shown). As *nrg1* ^*nc26*^ encodes a shorter truncation, we focused on characterizing this allele (Fig. S2).

### Nrg1 is dispensable for trabeculation, but is required for late juvenile and early adult fish to thrive

Our previous studies and others indicate that ErbB2 signalling is necessary to initiate cardiac trabeculation at 2 dpf 10. To evaluate whether loss of Nrg1 impairs trabeculation, we crossed *nrg1* ^*nc26*^ onto a *Tg(myl7:rasGFP)* transgenic background to label cardiomyocytes with a membrane‐targeted EGFP, then examined confocal optical cross sections of the ventricle. Interestingly, trabeculae emerged in *nrg1* ^*nc26*^ mutants and clutchmates between 2–3 dpf. To verify that trabeculation was not due to escape from requirement of ErbB2 signalling, we incubated embryos from 2 to 4 dpf with the ErbB2 tyrosine kinase specific inhibitor PD168393 38. While vehicle‐treated ventricles formed trabeculae, all genotypes had substantially reduced trabeculation after PD168393 treatment (Fig. 1F–G).

**Figure 2:**
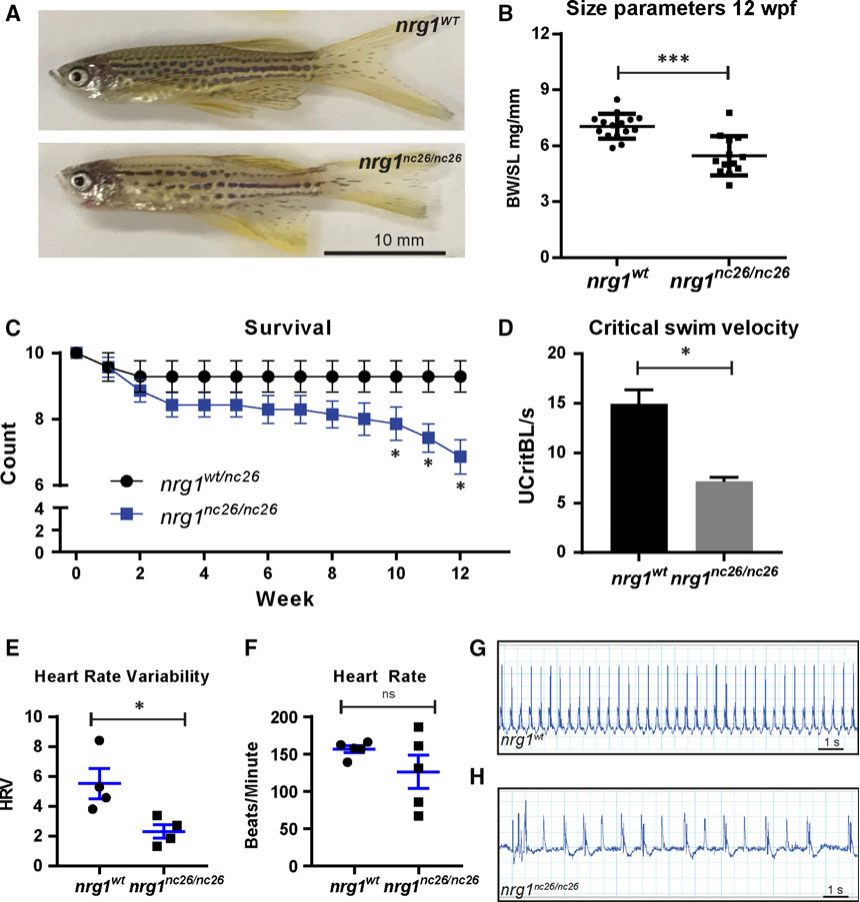
Adult cardiovascular consequences in *nrg1* ^*nc26*^ mutants. (**A**) Gross appearance of adult *nrg1* ^*WT*^ and *nrg1* ^*nc26/nc26*^ fish. (**B**) Body mass normalized to standard length in adult fish SL 20 ± 2, *N* = 12. (**C**) Critical swimming velocity of adult fish SL = 15 ± 1 *N* = 8. (**D**) Weekly survival of *nrg1* ^*WT*^ and *nrg1* ^*nc26/nc26*^ clutchmates reared separately in *N* = 7 tanks of 10 fish each. (**E, F**) Heart rate variance (HRV) and heart rate in beats per minute measured *via* electrocardiogram, SL = 15 ± 1, *N* = 3–5. (**G, H**) Representative electrocardiographs from *nrg1* ^*WT*^ and *nrg1* ^*nc26/nc26*^ fish. Statistical significance determined by t‐test, *ns* = not significant, *P* > 0.05, *P < 0.05, **P < 0.01, ***P < 0.001.

We next asked if *nrg1* is involved in other essential developmental processes. Consistent with its potential role in melanocyte development 39, 40, individual *nrg1* ^*nc26*^ mutant fish demonstrated defective pigmentation on genotypes encoding both striped (data not shown) and spotted patterning (Fig. 2A). When observed, adult mutants were typically smaller than age‐matched *nrg1* ^*wt*^ clutch mates and had reduced body mass in standard length‐matched clutch mates (Fig. 2B). We used the supernumerary neuromast phenotype to separate mutants from wild‐type or heterozygous clutch mates and evaluated survivorship at weekly intervals in a large, multi‐clutch cohort of fish. We observed that *nrg1* ^*nc26*^ mutant survival declines as early as 6 weeks of post‐fertilization (wpf) with significant reductions in survivorship by 10 wpf (Fig. 2C). To evaluate if the smaller size and early lethality may be attributable to reduction in physiological fitness, we assessed cardiovascular performance in size‐matched fish using a swim test. In support of reduced fitness, mutant fish showing no ostensible reduction in body mass failed to match *nrg1* ^*wt*^ maximum swim velocities (Fig. 2D). Interestingly, during routine handling, juvenile and adult mutants often failed to recover from anaesthesia with the benzocaine derivative Tricaine (MS222), suggesting they may suffer from cardiac arrhythmias. We used echocardiography to explore this possibility and found that there was no significant difference in heart rate. However, beats per minute were more variable in mutant fish. Heart rate variance scores were reduced in mutants compared to size‐matched controls (Fig. 2E–H). Together, these findings indicate that *nrg1* is involved in cardiovascular function.

### Cardiovascular malformations in pan‐Nrg1 mutants

**Figure 3:**
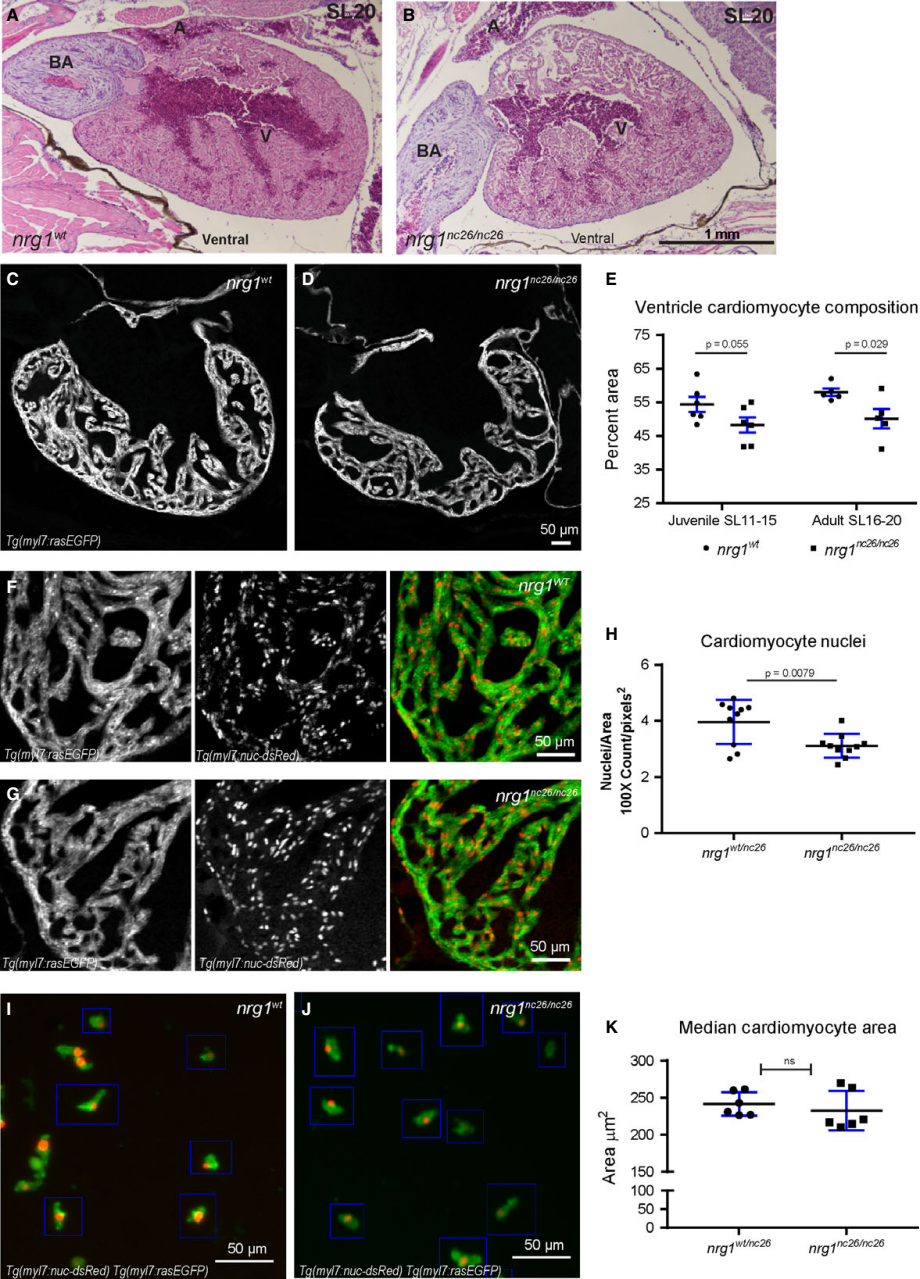
Reduced trabecular myocardium in *nrg1* ^*nc26*^ mutants due to lower cellularity. (A) Representative cross section of the heart in H&E‐stained section of formaldehyde‐fixed, paraffin‐embedded *nrg1* ^*WT*^ and *nrg1* ^*nc26/nc26*^ adult fish at SL = 20, *N* = 3. BA = Bulbous Arteriosus, V = Ventricle, A = Atrium. Scale bar 100 μm. (**C–E**) Intracardiac density measured from confocal 9.6 μm cross section of hearts from *Tg(myl7:rasGFP)* fish. (**C, D**) Representative images, SL = 13. (**E**) Intracardiac myocardial density quantified as the per cent area occupied by rasGFP+ cells in the outer curvature relative. (**F–H**) Cardiomyocyte numbers measured from confocal 9.6 μm cross section of hearts from double transgenic *Tg(myl7:rasGFP);Tg(myl7:nuc‐dsRED)* fish (**F, G**) Representative images, SL = 13. (**H**) Relative number of cardiomyocytes measured as from cross sections as the ratio of dsRED‐positive nuclei to rasGFP‐positive pixels in fish SL11‐SL20. (**I–K**) Cardiomyocyte size measured in mononuclear cells dissociated from *Tg(myl7:rasGFP);Tg(myl7:nuc‐dsRED)* hearts. (**I, J**) Representative dissociated cell preparations. (**K**) Median area of *N* > 200 cells per sample with 1–2 hearts per sample, *N* = 6 biological replicates.

The Neuregulin/ErbB pathway plays multiple, critical roles in cardiac development and homeostasis 5. To explore the possibility that structural abnormalities underlie the functional defect, we examined the heart in H&E‐stained sections from *nrg1* ^*nc26*^ fish and *nrg1* ^*WT*^ clutch mates showing minimal signs of cardiovascular distress at SL20 (Fig. 3A and B). These fish were chosen to help isolate the loss of Nrg1 as the driver of cardiovascular health defects as opposed to the consequences of declining health. Selecting unhealthy fish for heart examination could confound our results and fail to determine the role of Nrg1 loss. Although we did not observe consistent changes in overall chamber size, we observed a striking difference in the density of the myocardium. To further characterize this defect, we bred *nrg1* ^*nc26*^ fish onto a dual transgenic background to label myocardial cell borders with EGFP and nuclei with dsRed (*Tg(myl7:rasGFP);Tg(myl7:nuc‐dsRED*)), then measured myocardial density, nuclei count and size.

Examining confocal sections of these hearts, we measured intracardiac myocardial density as the area fraction occupied by cardiomyocytes within the outer third of the heart, and observed a strong trend towards reduced trabecular area in juvenile mutants and a significant reduction in adult mutants (Fig. 3C–E). As the vast majority of zebrafish cardiomyocytes are mononuclear 41, 42, we reasoned that myocardial area defects could arise from defects in the number and/or size of cardiomyocytes. We first asked if there were defects in the relative number of nuclei per surface area and found a significant reduction in the number of nuclei relative to myocardial area (Fig. 3F–H); zebrafish cardiomyocytes are mononuclear, so this reduction in nuclear number was proportional to a reduction in cell number. Next, we investigated whether these defects were accompanied by a reduction in cardiomyocyte size. To this end, we adapted a recently described method to dissociate zebrafish hearts into single‐cell preparations enriched for cardiomyocytes 34. We measured the surface area of 200–250 mononuclear cardiomyocytes from each sample and did not observe significant differences in either the mean or median surface area (data not shown, Fig. 3I–K). Due to variability in dissociation efficiencies between experiments, the absolute number of cardiomyocytes in each heart could not be formally addressed. Together, these findings suggest that the reduced intracardiac surface area is largely due to there being fewer cells rather than smaller cells in the heart.

### Nrg1‐III regulates larval cardiac nerve plexus development

**Figure 4:**
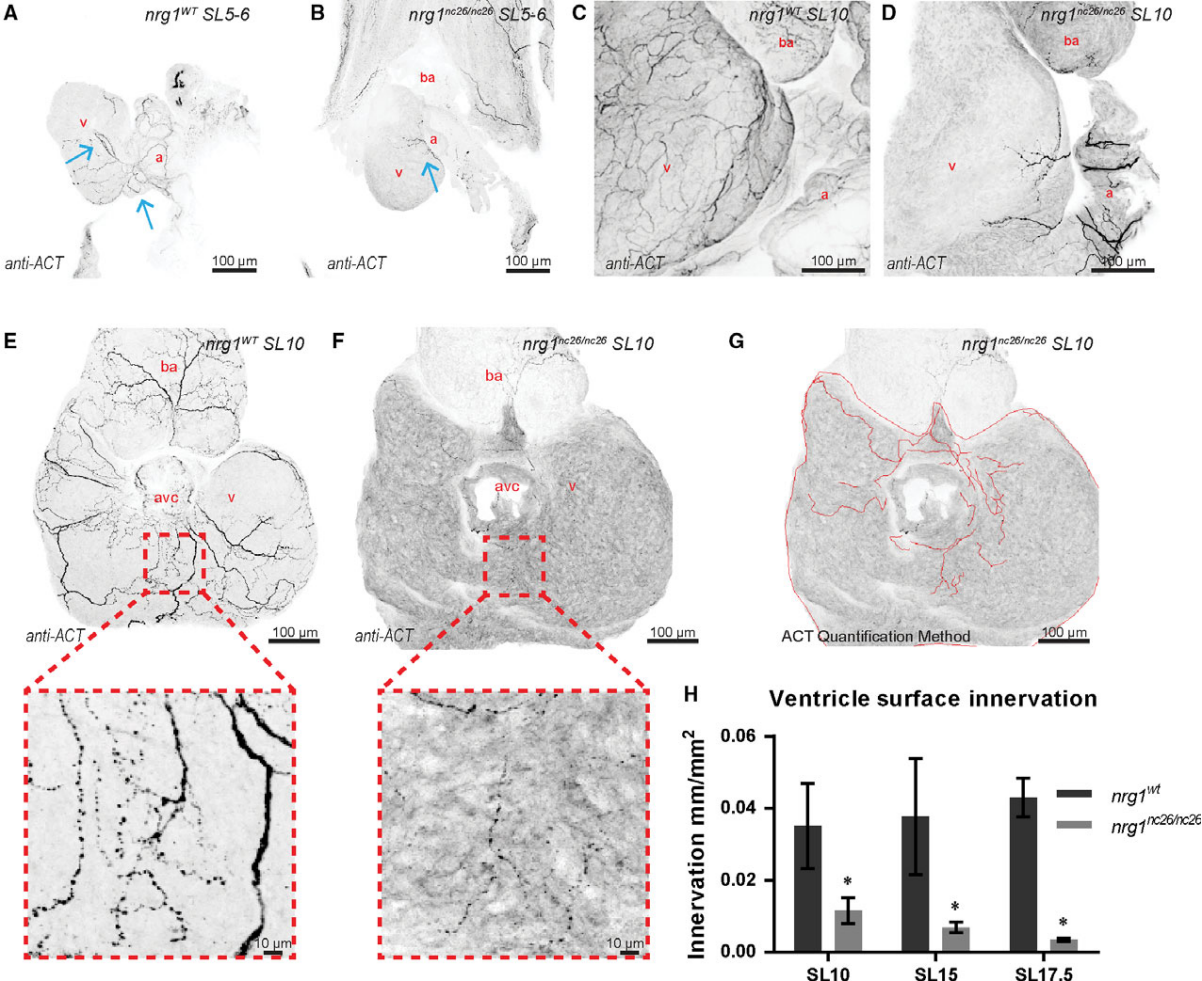
Reduced ventricle surface innervation from juvenile to adult stages. (**A–F**) Representative z‐projections of confocal images of axons stained with anti‐acetylated α‐tubulin. (**A, B**) Larvae at SL 5–6 were fixed and stained *in situ* with the heart partially dissected. (**C, D**) Juvenile hearts at SL 10 demonstrated partial atrial and limited ventricular innervation in *nrg1* ^*nc26/nc26*^ hearts. (**E, F**) Maximum intensity, z‐project of confocal images of dorsal surface of ventricle with atrium removed at SL10 with magnified view of representative innervated regions. (**G, H**) Quantification of ventricle surface innervation using methodology illustrated in (**G**) as the quotient of the total length of axons and ventricle surface. (**H**) Innervation quantified in *N* > 3 hearts at SL 10 ± 1, SL 15 ± 1 and SL 17.5 ± 1. Abbreviations a = atrium, v = ventricle, ba = bulbous arteriosus. Scale bars are 100 μm. Student's t‐test mutant compared to wild‐type. Error bars are S.E.M. *P = 0.01–0.05. Blue arrows point to axons.

Given the presence of supernumerary neuromasts, heart rate variance, and failure to revive from tricaine, we postulated that loss of *nrg1* might lead to defects in formation of the cardiac nerve plexus. Previous studies suggest that the timing of late cardiac development events in zebrafish, such as coronary vessel formation, correlate well with fish age and standard length 43. To test this hypothesis, we thus isolated hearts from juvenile fish at standard lengths (SL) spanning larval, juvenile, and adult life stages and visualized axons using an antibody against acetylated α‐tubulin (ACT). At SL 5–6, wild‐type hearts examined *in situ* demonstrated robust atrial innervation and variable indications of ventricular innervation emanating from the atrioventricular canal (AVC) (Fig. 4A and B). In contrast, at this stage and at SL 10, all ventricles examined from *nrg1* ^*nc26*^ mutant fish were largely devoid of axons with limited atrial innervation (Fig. 4A–D). Interestingly, the nerve plexus appears to project from the atrium, through the AVC, then across the ventricle surface. This is in agreement with a recent study examining adult cardiac innervation *en bloc* 21. To quantify this apparent reduction in innervation while capturing emerging innervation, the atrium was detached and dorsal surface of the ventricle was imaged, centering on the AVC (Fig. 4E and F). Indeed, *nrg1* ^*nc26*^ axons rarely covered more than a small fraction of the dorsal surface and were dramatically reduced compared to size‐matched controls (Fig. 4G and H). These data indicate that *nrg1‐*deficiency leads to a lack of innervation on the ventricle surface that is associated with long‐term survival consequences.

We next asked which isoform(s) of Nrg1 are associated with this phenotype. In previous studies, we show that while *nrg1‐I* is the primary isoform expressed in the heart, both *nrg1‐I* and *nrg1‐II* are dispensable for development 27. *nrg1‐III* is primarily expressed in neuronal tissue with known roles in regulating myelination of developing nerves. If the defects associated with loss of *nrg1* are primarily attributable to the type‐III isoform, *nrg1‐III*, then we would expect that *nrg1* ^*z26*^ mutants would have a similar phenotype as *nrg1* ^*nc26*^ and *nrg1* ^*nc27*^ mutants. We repeated key components of this study in *nrg1* ^*z26*^ mutants and observed similar defects in total cardiac performance, electrocardiac abnormalities and reduced cardiac innervation, although the *nrg1* ^*z26*^ mutants exhibited a less significant survival defects compared to *nrg1* ^*nc26*^ mutants (Fig. S3A–H). Our findings suggest that Nrg1 is required for cardiovascular performance through *nrg1‐III*‐dependent mechanisms that regulating the formation of cardiac nerve plexus.

## Discussion

In addition to its crucial role during cardiac development, the Nrg1/ErbB system is important for adult cardiovascular homeostasis 4 Promising clinical trials have demonstrated that recombinant NRGs may become a new class of cardiovascular therapies that directly promote reverse remodelling in heart failure 23. In the light of this, a better understanding of the potential consequences of manipulations in the Nrg1/ErbB2 signalling pathway could be informative.

Previous reports in mammalian systems strongly indicated that Nrg1 is critical for certain aspects of both nervous system and cardiovascular development; however, placental development in mammals precludes direct evaluation. To circumvent this limitation, we used zebrafish as a genetically tractable and relatively hypoxia tolerant model organism. Nrg1 is part of a large family of EGF‐like ligands and the EGF‐like domain is important for binding to ErbB tyrosine kinase receptors. Nrg1 is widely considered the primary ligand for cardiac‐expressed ErbB2/ErbB4 heterodimers. Nrg1 binding to the kinase‐dead ErbB2 stimulates tyrosine phosphorylation in the obligate heterodimer ErbB4, which in turn has no known ligand‐binding affinity 3, 5, 7. Notably, ErbB2 can also heterodimerize with ErbB3, which is canonically found in neuronal systems. Although ErbB2 kinase activity is essential for zebrafish heart, fin and lateral line development, little is known about the genetic requirement for Nrg1 in zebrafish heart development. One of the challenges to understanding Nrg1 biology is that mouse, human and zebrafish *nrg1* are all alternatively spliced, and previous studies have shown differential requirements for *nrg1* isoforms in distinct cell and tissue‐specific functions 4 Alternative splicing of *nrg1* produces three main isoforms, *nrg1‐I*,* nrg1‐IIa‐c* and *nrg1‐III*, which differ primarily in their N‐terminal sequence and share EGF‐like, transmembrane, and ‘neuregulin’ domains^7^, ^37^. In previous study, we showed that while *nrg1‐I* is the primary isoform expressed in the heart, but both *nrg1‐I* and *nrg1‐II* are dispensable for development 27. Instead, a recent report described an essential role for *nrg2a* in zebrafish heart development, in particularly 44. *nrg1‐III* is primarily expressed in neuronal tissue with known roles in regulating myelination of developing nerves 8, 10, 19.

Here, we used CRISPR/Cas9 gene targeting to generate *nrg1* null alleles, *nrg1* ^*nc26*^ and *nrg1* ^*nc27*^, that target all known isoforms of *nrg1* and explored the cardiovascular consequences of total loss of this gene across zebrafish life stages (embryonic, larval, juvenile and adult). By targeting all alleles, we aimed to clarify whether there are any roles for this gene, which is so critical for development in mammalian systems, in zebrafish cardiovascular development and homeostasis. Consistent with our previous report, we did not observe defects in the initial development of cardiac trabeculae in *nrg1* ^*nc26/nc26*^ embryos or larvae. In pilot studies, siblings of all genotypes were reared in the same tank. Zebrafish growth is primarily limited by food availability and competition between individuals housed within the same tank. By late juvenile stages, *nrg1* ^*nc26/nc26*^ individuals were much smaller than their tank mates, suggesting a deficiency in fitness. Mutant larvae were indistinguishable from wild‐type and heterozygous siblings except for an increase in neuromasts in the lateral line. These supernumerary neuromasts were detectable in living larvae by a vital dye, allowing separate rearing of the genotypes. Despite separate rearing to eliminate intra‐tank competition, early mortality occurred in *nrg1* ^*nc26/nc26*^ fish at late juvenile to early adult stages.

*Nrg1* ^*nc26/nc26*^ mutants had major deficiencies in the cardiac nerve plexus that emerge in larval stages at approximately SL = 5 mm. Histological analysis of *nrg1* ^*nc26/nc26*^ mutant hearts from SL 10–15 and SL 16–20 suggests that underlying structural defects, particularly in intracardiac myocardial density, may contribute to this decline as zebrafish mature. It is important to note intracardiac myocardial density did not show significant reduction in Nrg1 mutants until SL 16–20. Swimming performance is considered a general indication of performance capacity in fish. Therefore, we used the swimming assay to establish relative baseline measurements of health and fitness in both our wild‐type and mutant zebrafish. Nerve plexus reduction, which began in larval stages of mutants and continued throughout maturation, was correlated with decreased swimming performance and heart rhythm abnormalities in adulthood. Our data suggest that malformation of the cardiac nerve plexus which normally extends over the surface of the ventricle and likely aids in coordinated contraction either underlies or contributes to cardiovascular functional defects and eventual mortality. In combination with recent reports describing the importance of nerves in zebrafish cardiac regeneration, this study adds to a growing body of literature supporting essential conserved roles for crosstalk between nerves and the heart in during development and homeostasis.

If the defects associated with loss of *nrg1* are primarily attributable to the type‐III isoform, *nrg1‐III*, then we would expect that *nrg1* ^*z26*^ mutants should have a similar phenotype as *nrg1* ^*nc26*^ and *nrg1* ^*nc27*^ mutants. Indeed, we repeated key components of this study in *nrg1* ^*z26*^ mutants and observed similar defects in total cardiac performance, electrocardiac abnormalities and reduced cardiac innervation. However, *nrg1* ^*z26*^ mutants did not have the same survival defects. This difference could be attributable to the fact that *nrg1* ^*z26*^ allele produces a protein, albeit one with reduced function, while the *nrg1* ^*nc26*^ allele does not appear to produce any protein product. Perhaps due to retention of other domains in this loss of function mutant. Together, these data indicate that *nrg1,* specifically through *nrg1‐III*, is an essential regulator of cardiac nerve plexus formation.

Although a great deal has been learned about the role of Nrg1 and ErbB receptors in the cardiovascular system, there are many important unanswered basic biology questions related to Nrg/ErbB signalling. Before we will be able to utilize the full potential of Nrg1‐based therapy and tailor it to patients, we must untangle the mechanisms through which Nrg1 causes the observed effects on homeostatic and cardiac functions. This knockout study of Nrg1 signalling provides new insights into how Nrg1 may contribute to proper cardiac innervation, which in turn promotes cardiac maturation and function. An in‐depth knowledge of the effects of Nrg1 on the many cell types, both cardiovascular and nervous, is paramount to the development of therapeutics that fully harness the beneficial effects of this pathway.

## Author contributions

D.B. and L.A. designed and performed the majority of experiments, analyzed data and wrote the manuscript. C.I. and H. M. performed the experiments and analyzed data. R.A. provided intellectual input and edited the manuscript. L. Q. and J. L. provided intellectual input and supervised the work. All authors commented on the manuscript.

## Conflict of interest

The authors confirm that there are no conflict of interests.

## Supporting information


**Fig. S1** Generation and validation of *nrg1* ^*nc27*^ allele. (A) CRISPR/Cas9 targeting at Exon 6 produced the *nrg1* ^*nc27*^ allele with an eight amino acid deletion. (B) Sanger sequence of *nrg1* ^*nc27*^ allele. (C) Representative *nrg1* ^*wt*^ and *nrg1* ^*nc26/nc26*^ clutchmates stained with Mitotracker Red to detect neuromasts in the developing lateral line at 4‐5 dpf. Red arrows designate neuromasts. (C’) Relative frequency of the number of neuromasts counted in embryos from heterozygous inbreedings of *nrg1* ^*wt/nc26*^ fish.

**Fig. S2** Predicted translation of *nrg1‐I*. Predicted translation of *nrg1‐I* mRNA from *nrg1* ^*wt*^ *, nrg1* ^*nc26*^, and *nrg1* ^*nc27*^ alleles.

**Fig. S3** Adult cardiovascular consequences in *nrg1* ^*z26*^, *nrg1‐III* specific allele. (A) Gross appearance of adult *nrg1* ^*WT*^ and *nrg1* ^*z26/z26*^ fish. (B) Body mass normalized to standard length in adult fish SL 20±2, *N*=12. (C) Critical swimming speed of adult fish SL=15±1 *N*=8. (D) Weekly survival of *nrg1* ^*WT*^ and *nrg1* ^*z26/z26*^ clutchmates reared separately in *N*=7 tanks of 10 fish each. (E‐F) Heart rate variance (HRV) and heart rate in beats per minute measured *via* electrocardiogram, SL=15±1, *N*=3–5. (G‐H) Representative electrocardiographs from *nrg1* ^*WT*^ and *nrg1* ^*z26/z26*^ fish. (I) Representative z‐projections of confocal images anti‐acetylated α‐tubulin axon staining on the dorsal surface of SL 15 fish with the atrium removed. (J) Quantification of ventricle surface innervation as the quotient of the total length of axons and ventricle surface in *N*>3 hearts at SL 10±1, SL 15±1, and SL 17.5±1. Abbreviations a=atrium, v=ventricle, ba= bulbous arteriosus. Student's *T*‐test mutant compared to wild type. Error bars are S.E.M. ^*P*=0.05‐0.10, *P=0.01‐0.05, **P=0.001–0.01.
