# Supplementary figures and images for: Neuregulin‐1 is essential for nerve plexus formation during cardiac maturation
Source: J Cell Mol Med. 2017 Dec 19;22(3):2007–17. doi: 10.1111/jcmm.13408 (PMC5824398; doi:10.1111/jcmm.13408)

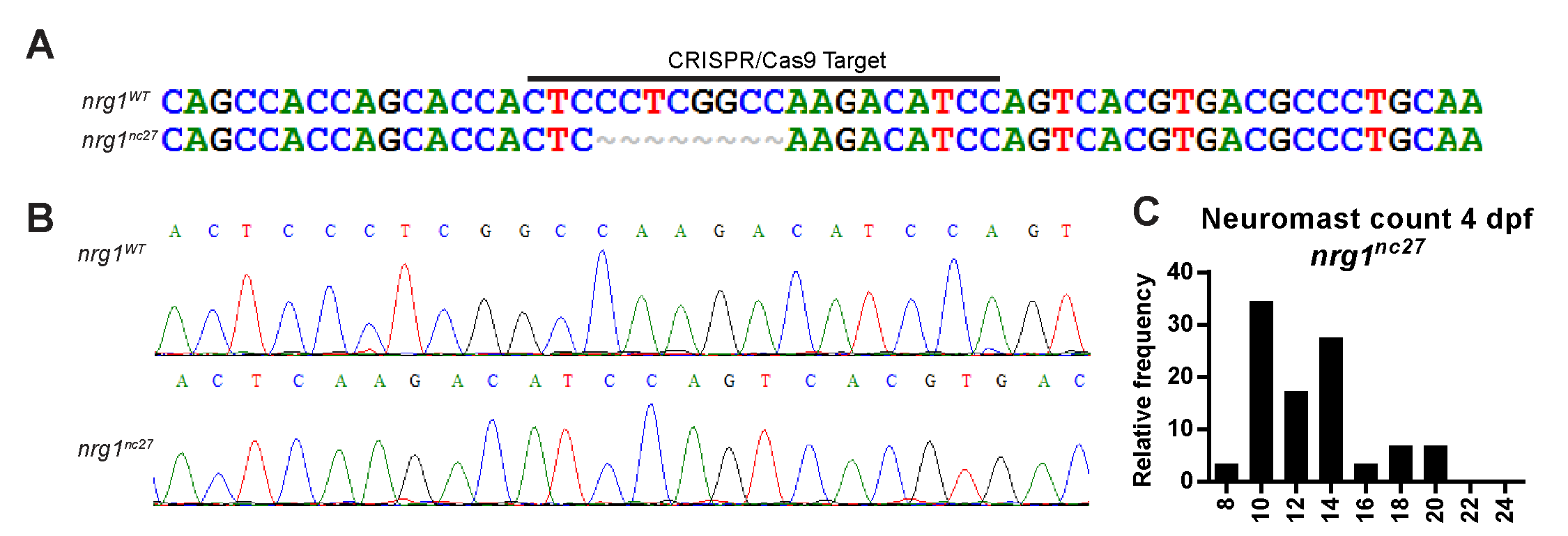

Supplement: Supplementary file 1 — Fig. S1 Generation and validation of nrg1 nc27 allele. (A) CRISPR/Cas9 targeting at Exon 6 produced the nrg1 nc27 allele with an eight amino acid deletion. (B) Sanger sequence of nrg1 nc27 allele. (C) Representative nrg1 wt and nrg1 nc26/nc26 clutchmates stained with Mitotracker Red to detect neuromasts in the developing lateral line at 4‐5 dpf. Red arrows designate neuromasts. (C’) Relative frequency of the number of neuromasts counted in embryos from heterozygous inbreedings of nrg1 wt/nc26 fish. [file JCMM-22-2007-s001.tif]

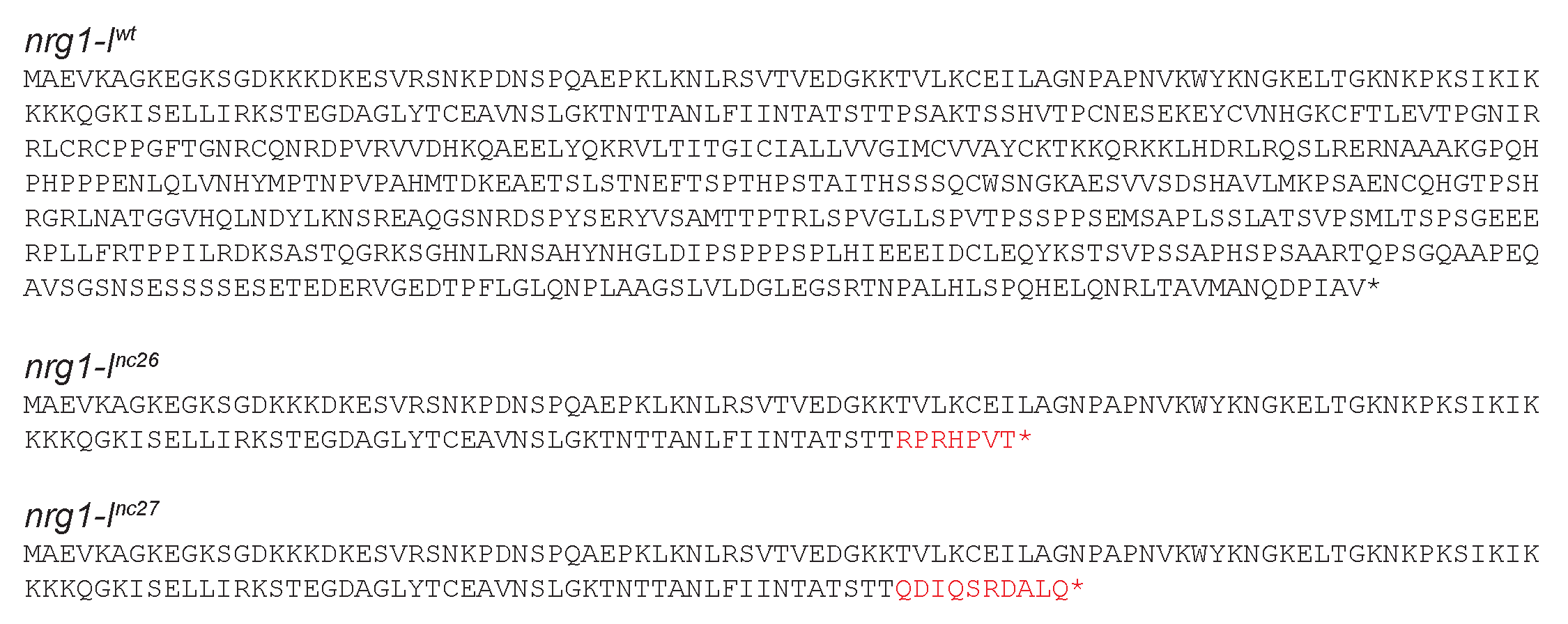

Supplement: Supplementary file 2 — Fig. S2 Predicted translation of nrg1‐I. Predicted translation of nrg1‐I mRNA from nrg1 wt , nrg1 nc26, and nrg1 nc27 alleles. [file JCMM-22-2007-s002.tif]

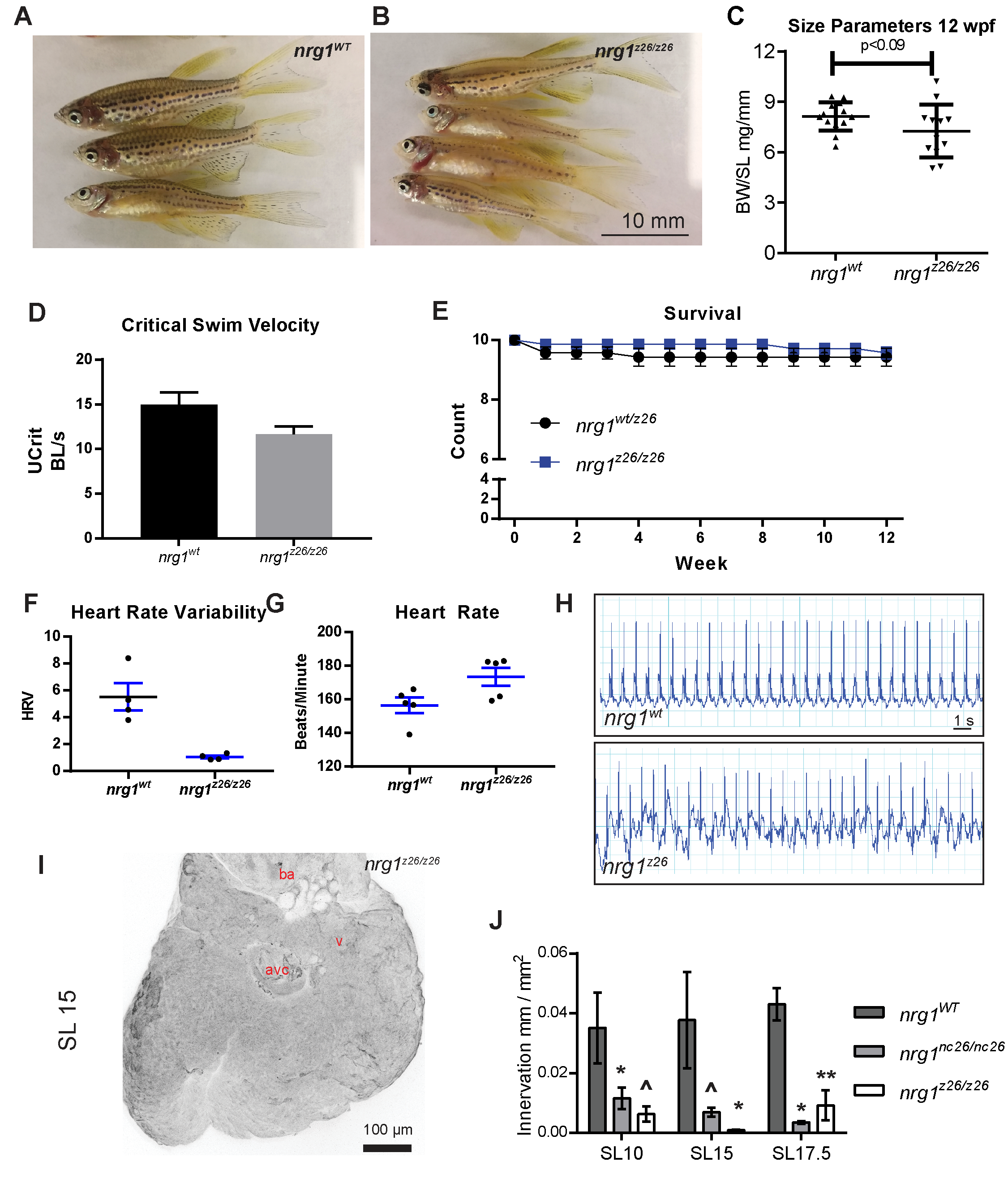

Supplement: Supplementary file 3 — Fig. S3 Adult cardiovascular consequences in nrg1 z26, nrg1‐III specific allele. (A) Gross appearance of adult nrg1 WT and nrg1 z26/z26 fish. (B) Body mass normalized to standard length in adult fish SL 20±2, N=12. (C) Critical swimming speed of adult fish SL=15±1 N=8. (D) Weekly survival of nrg1 WT and nrg1 z26/z26 clutchmates reared separately in N=7 tanks of 10 fish each. (E‐F) Heart rate variance (HRV) and heart rate in beats per minute measured via electrocardiogram, SL=15±1, N=3–5. (G‐H) Representative electrocardiographs from nrg1 WT and nrg1 z26/z26 fish. (I) Representative z‐projections of confocal images anti‐acetylated α‐tubulin axon staining on the dorsal surface of SL 15 fish with the atrium removed. (J) Quantification of ventricle surface innervation as the quotient of the total length of axons and ventricle surface in N>3 hearts at SL 10±1, SL 15±1, and SL 17.5±1. Abbreviations a=atrium, v=ventricle, ba= bulbous arteriosus. Student's T‐test mutant compared to wild type. Error bars are S.E.M. ^P=0.05‐0.10, *P=0.01‐0.05, **P=0.001–0.01. [file JCMM-22-2007-s003.tif]
