# Supplementary material for: Neuregulin‐1 is essential for nerve plexus formation during cardiac maturation
Source: J Cell Mol Med. 2017 Dec 19;22(3):2007–17. doi: 10.1111/jcmm.13408 (PMC5824398; doi:10.1111/jcmm.13408)
Supplement: Supplementary file 4 [file JCMM-22-2007-s004.docx]

**Figure S1. Generation and validation of *nrg1^nc27^* allele**

(A) CRISPR/Cas9 targeting at Exon 6 produced the *nrg1^nc27^* allele with an eight amino acid deletion. (B) Sanger sequence of *nrg1^nc27^* allele. (C) Representative *nrg1^wt^* and *nrg1^nc26/nc26^* clutchmates stained with Mitotracker Red to detect neuromasts in the developing lateral line at 4-5 dpf. Red arrows designate neuromasts. (C’) Relative frequency of the number of neuromasts counted in embryos from heterozygous inbreedings of *nrg1^wt/nc26^* fish.

**Figure S2. Predicted translation of *nrg1-I***

Predicted translation of *nrg1-I* mRNA from *nrg1^wt^, nrg1^nc26^*, and *nrg1^nc27^* alleles.

**Figure S3. Adult cardiovascular consequences in *nrg1^z26^*, *nrg1-III* specific allele**

(A) Gross appearance of adult *nrg1^WT^* and *nrg1^z26/z26^* fish*.* (B) Body mass normalized to standard length in adult fish SL 20±2, N=12. (C) Critical swimming speed of adult fish SL=15±1 N=8. (D) Weekly survival of *nrg1^WT^* and *nrg1^z26/z26^* clutchmates reared separately in N=7 tanks of 10 fish each. (E-F) Heart rate variance (HRV) and heart rate in beats per minute measured via electrocardiogram, SL=15±1, N=3-5. (G-H) Representative electrocardiographs from *nrg1^WT^* and *nrg1^z26/z26^* fish. (I) Representative z-projections of confocal images anti-acetylated α-tubulin axon staining on the dorsal surface of SL 15 fish with the atrium removed. (J) Quantification of ventricle surface innervation as the quotient of the total length of axons and ventricle surface in N>3 hearts at SL 10±1, SL 15±1, and SL 17.5±1. Abbreviations a=atrium, v=ventricle, ba= bulbous arteriosus. Student’s T-test mutant compared to wild type. Error bars are SEM. ^p=0.05-0.10, * p=0.01-0.05, ** p=0.001-0.01.
